# Dewdrop Metasurfaces and Dynamic Control Based on Condensation and Evaporation

**DOI:** 10.1002/advs.202404010

**Published:** 2024-08-21

**Authors:** Runqi Jia, Yongxin Jing, Hongchen Chu, Ruwen Peng, Mu Wang, Yun Lai

**Affiliations:** ^1^ National Laboratory of Solid State Microstructures School of Physics and Collaborative Innovation Center of Advanced Microstructures Nanjing University Nanjing 210093 China; ^2^ School of Physics and Technology Nanjing Normal University Nanjing 210023 China

**Keywords:** dewdrop condensation, tunable metasurface, ultra‐broadband absorption

## Abstract

Dewdrops, the droplets of water naturally occurring on leaves and carapaces of insects, are a fascinating phenomenon in nature. Here, a man‐made array of dewdrops with arbitrary shapes and arrangements, which can function as an electromagnetic metasurface, is demonstrated. The realization of the dewdrop array is enabled by a surface covered by a tailored pattern of hydrophilic and hydrophobic coatings, where tiny droplets of water can aggregate and form dewdrops on the former. Interestingly, this metasurface made of dewdrops can be modulated by the condensation and evaporation process. By increasing relative humidity and decreasing temperature, the dewdrop metasurface is gradually formed with increasing amounts of water. While the reverse operation can make it completely disappear. This idea is demonstrated through two examples with different functions of dynamically controllable microwave absorption and scattering. The work shows a principle to construct functional electromagnetic devices with dewdrops, as well as a mechanism of dynamic control based on condensation and evaporation, promising unprecedented applications.

## Graphical abstract

This study utilizes tailored patterns of hydrophilic and hydrophobic coatings to realize a novel concept: dewdrop metasurfaces, which are man‐made arrays of dewdrops with arbitrary shapes and arrangements. Dewdrop metasurfaces enable dynamic control based on condensation and evaporation processes for the first time, opening up a convenient and environmentally friendly approach for real‐time manipulation of electromagnetic wave absorption and scattering.

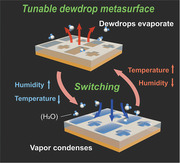

## 1 Introduction

Metasurfaces, the planar artificial materials composed of sub‐wavelength structures, have attracted an increasing amount of interest in recent years. By inducing the predesigned resonance of electromagnetic (EM) waves with metallic or dielectric sub‐wavelength structures,^[^ ^1^, ^2^, ^3^, ^4^, ^5^ ^]^ metasurfaces exhibit the capability to conveniently manipulate EM waves.^[^ ^6^, ^7^, ^8^ ^]^ Traditional passive metasurfaces pose limitations in their singular and fixed functionality. Thus recently, there has been a dramatically increasing demand for dynamically controllable metasurfaces, aiming to enhance their adaptability to diverse scenarios, e.g., electrically tunable metasurfaces,^[^ ^9^, ^10^, ^11^, ^12^, ^13^ ^]^ mechanically tunable metasurfaces,^[^ ^14^, ^15^, ^16^, ^17^, ^18^, ^19^ ^]^ optically switchable metasurfaces,^[^ ^20^, ^21^, ^22^ ^]^ thermal or chemically switchable metasurfaces,^[^ ^23^, ^24^, ^25^, ^26^, ^27^, ^28^ ^]^ etc. Dynamic metasurfaces with thermal modulation have been mostly based on the integration of thermally sensitive materials, such as phase‐change materials^[^ ^29^, ^30^ ^]^ and liquid crystals.^[^ ^31^, ^32^, ^33^ ^]^

**Figure 1:**
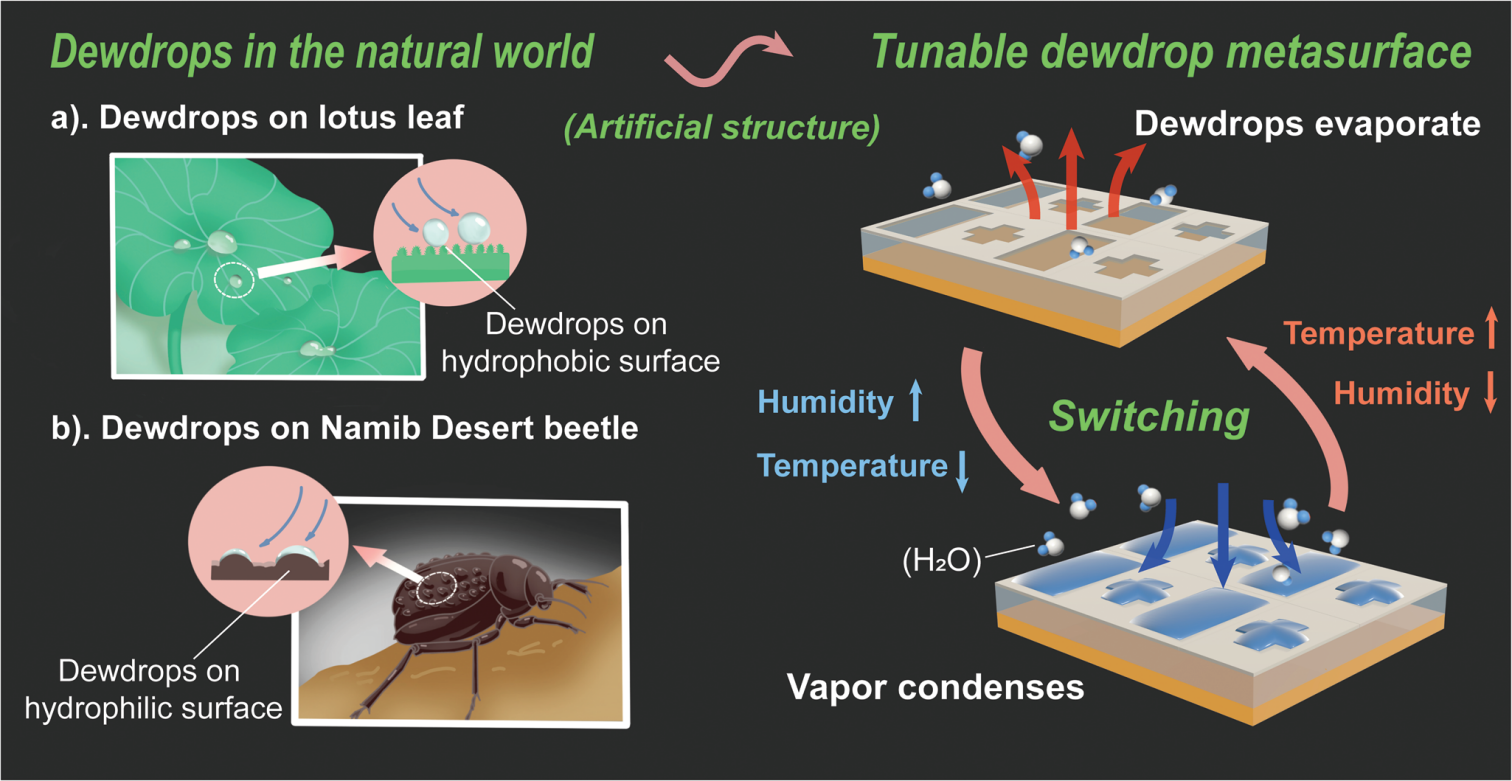
Schematic diagram of dewdrops in nature and tunable dewdrop metasurfaces. The states of the dewdrop metasurfaces can be modulated by the substrate surface properties as well as by the environmental temperature and relative humidity.

Condensation and evaporation describe the transformation of material states between liquid and gas, which can also be controlled by thermal modulation. However, so far, dynamic control of artificial materials based on these processes has been seldom discussed. Notably, dewdrops, the droplets of water, are a ubiquitous dielectric material with exceptional fluidity and remarkable EM response characteristics in both microwave^[^ ^34^, ^35^, ^36^, ^37^, ^38^, ^39^ ^]^ and optical spectra.^[^ ^40^ ^]^ Within the natural realm, animals and plants adeptly harness environmental factors and their inherent surface wettability to facilitate the condensation and gathering of dewdrops, which are important to their survival, such as the lotus leaves and desert beetles^[^ ^41^ ^]^ shown in **Figure** **1**. The manipulation of dewdrops, orchestrated through alterations in both environmental conditions and surface attributes, is widespread in nature. So far, many research topics based on water droplets have been focused on the morphology^[^ ^42^ ^]^ and dynamic mechanisms^[^ ^43^ ^]^ of droplets on artificial surfaces, which facilitate the development of microfluidics.^[^ ^44^ ^]^ Especially, the condensation process of vapor on artificial surfaces with hydrophilic or hydrophobic properties has been extensively explored, yielding applications, such as highly efficient heat transfer surfaces, self‐cleaning, antifogging, and antifrosting.^[^ ^45^, ^46^ ^]^ Nevertheless, the concept of establishing a real‐time controlled environment for the condensation and evaporation of dewdrops, aiming to create a customizable and dynamic metasurface, remains unexplored to this day.

In this work, we demonstrate tunable dewdrop metasurfaces (TDMs) that can manipulate microwave absorption and reflectance based on real‐time controlled condensation and evaporation as shown in Figure 1. The TDMs are man‐made arrays of dewdrops realized on a dielectric surface. The shapes and arrangements of the dewdrops can be controlled by coating the surface with a designed pattern of hydrophilic and hydrophobic materials. Since water exhibits large relative permittivity in the microwave regime as explained in the Supporting Information, Figure S1, the EM response of the dewdrops can be tailored by engineering their geometric parameters, which is similar to the case of metallic structures. When the metasurface is cooled and the ambient humidity increases, dewdrops condense on the predesigned substrate. The structure of the metasurface changes due to the formation of dewdrops. In contrast, cessation of cooling and dehumidification result in the evaporation of the dewdrops, thereby altering the structural state of the dewdrop metasurface. Through real‐time control of ambient temperature and relative humidity, flexible regulation of the presence and disappearance of dewdrop metasurfaces is achieved, along with their functionalities on microwave absorption and wavefront modulation. Our work thus successfully verifies the feasibility of artificial materials with dynamic control using condensation and evaporation.

## 2 Results

### 2.1 Constructing TDMs Through Condensation and Evaporation

**Figure 2:**
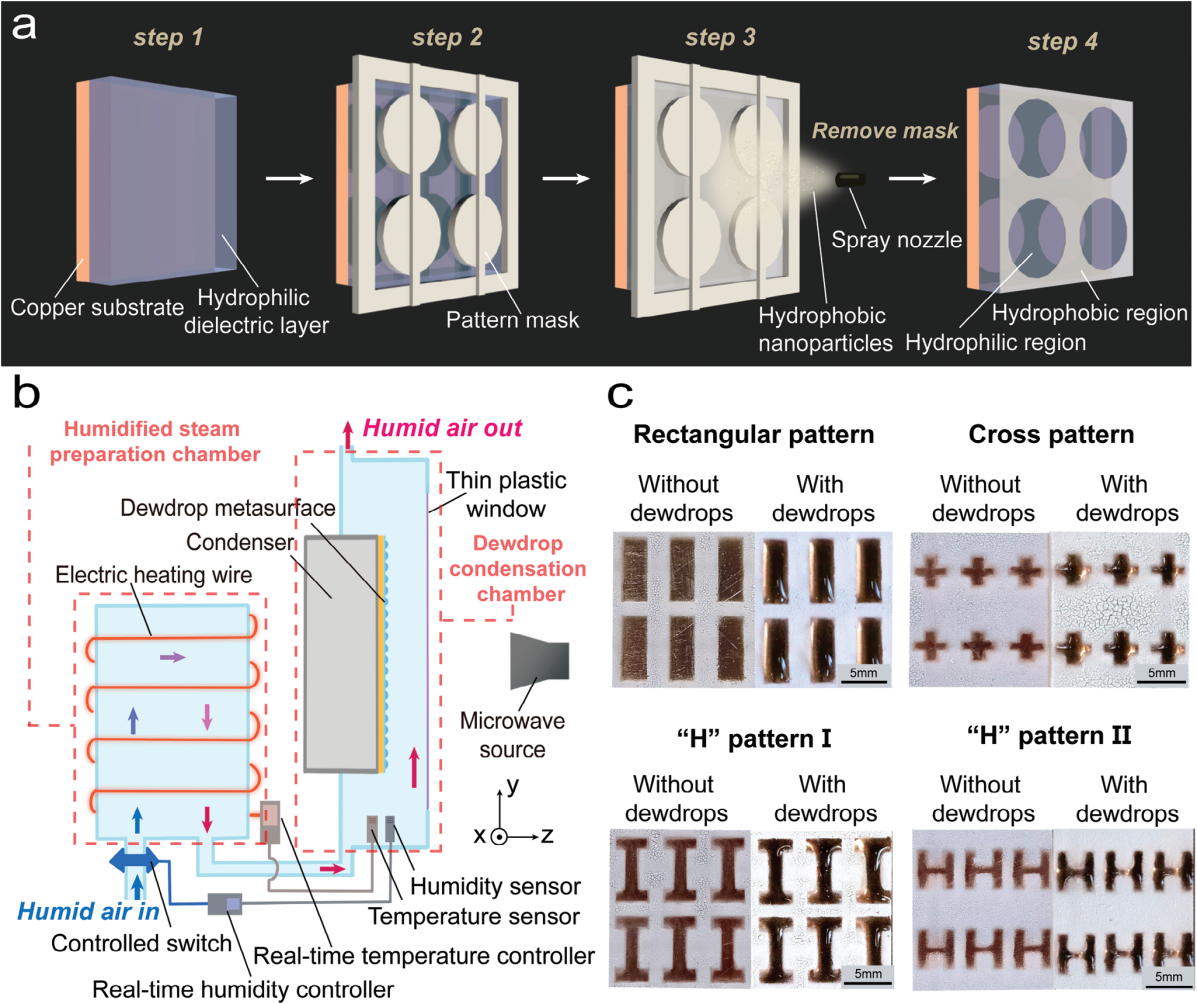
Preparation of dewdrop metasurfaces and principle of unit cell manufacture. a) Fabrication processes of dewdrop metasurface using hydrophobic nanoparticles spray coating treatment. The pattern mask blocks hydrophobic nanoparticles from depositing on hydrophilic patterns. b) Side view of real‐time controlled dewdrop condensation and evaporation devices. c) Various dewdrop patterns condensed on hydrophilic/hydrophobic hybrid dewdrop metasurfaces, and all of the scale bars represent 5 mm.

To achieve TDMs through the condensation process of water vapor, one critical step is to generate plump dewdrop patterns on a flat substrate during the condensation procedure. The condensation of water vapor into dewdrops requires relative humidity to reach the dew point and nucleation sites on the condensation surface.^[^ ^47^, ^48^ ^]^ Meanwhile, the wettability of a substrate surface determines the condensation type,^[^ ^49^ ^]^ the morphology of dewdrops,^[^ ^42^, ^50^, ^51^, ^52^ ^]^ and the nucleation rate.^[^ ^53^ ^]^ For a hydrophilic surface with relatively higher surface energy, the condensation type is observed as film condensation^[^ ^49^ ^]^ where the contact angle (the angle between a liquid surface and a solid surface where they meet) is sharp. The initial dewdrops tend to coalesce with adjacent droplets and change the growth morphology from spherical to island‐like irregular droplets, and finally, to plump large dewdrops.^[^ ^54^ ^]^ However, the hydrophobic surface has lower surface energy, and the type of condensation is dropwise condensation, which results from an obtuse contact angle. The morphology of dewdrops on hydrophobic surfaces remains nearly unchanged, consistently maintaining a spherical shape.^[^ ^55^ ^]^ Besides, according to nucleation theory, the hydrophilic surface has a higher initial nucleation density, which means the initial coalescence and growth of dewdrops occur more readily.^[^ ^53^, ^56^ ^]^ In order to verify the condensation performance on surfaces of different wettability, we have tested the volume of condensate droplets on hydrophobic and hydrophilic regions. We find that, for hydrophilic–hydrophobic hybrid patterns in our experiments, the total volume of dewdrops on hydrophobic regions was significantly lower than that on hydrophilic regions under the same condensation duration (see the Supporting Information, Table S1). Therefore, the dewdrop pattern of a TDM can be realized by engineering the distribution of distinctive wettability on the substrate surface. **Figure** **2**a schematically shows the fabrication processes of a hydrophilic pattern. The substrate of a TDM is composed of a thin copper layer and a hydrophilic dielectric layer. The front surface of the dielectric layer is used to generate hydrophilic patterns that support dewdrops. Such a substrate surface is homogeneously hydrophilic (e.g., SiO_2_ glass (SiO_2_ ≥ 99.9%); soda‐lime glass (SiO_2_ ≈ 60%–75%, Na_2_O ≈ 10%–25%, Ca_2_O ≈ 5%–15%; 95ceramic (Al_2_O_3_ ≥ 95%)). A complementary hydrophobic pattern can be coated on this surface by covering a patterned mask and then spray painting it with hydrophobic nanoparticles. The component of the hydrophobic layer is Fluorosilane polymer.^[^ ^57^, ^58^ ^]^ Areas concealed by the mask remain hydrophilic. On the contrary, uncovered regions exposed to the spray are coated with hydrophobic nanoparticles, creating an extremely thin, grayish‐white hydrophobic layer with a thickness of around 15 µm. After removing the patterned mask, a surface with periodic patterns of hydrophilic and hydrophobic regions is obtained, which can be utilized for the subsequent condensation of dewdrops of desired patterns. The detailed fabrication process and materials involved in hybrid wettability surface manufacture are illustrated in the Supporting Information, Figure S2.

In order to realize real‐time control over the dewdrop unit cells on the metasurface, we have designed the dewdrop preparation device as illustrated in Figure 2b. This apparatus comprises two distinct functional chambers: a humidified steam preparation chamber heated by electrical heating wire, and a connected dewdrop condensation chamber housing both a thermostatic condenser and the metasurface substrate we processed. The temperature (relative humidity) information of the humid air detected by the temperature (humidity) sensor is transmitted to the temperature (relative humidity) controller. The controller can automatically regulate the operational status of the electric heating wire and the vapor switch to readjust the vapor parameters to guarantee tunable and stable relative humidity and temperature. Therefore, tunable and stable morphologies of dewdrop unit cells can be achieved. In the dewdrop condensation chamber, the real‐time controlled hot vapor would condense on the cold blank metasurface substrate. Periodic dewdrop structures emerge on hydrophilic regions and can be observed through the thin plastic window of the condensation chamber. The picture of real‐time controlled dewdrop condensation device is presented in the Supporting Information, Figure S3. Figure 2c illustrates dewdrop unit cells with different morphologies obtained through the above‐mentioned dewdrop metasurface preparation method. When the relative humidity is increased, dewdrops tend to condense in the hydrophilic regions due to the distinctive wetting properties of the hydrophilic and hydrophobic regions. Thanks to the fluid nature of water, dewdrops transform from conventional spherical‐cap shape to the shape of the hydrophilic region when the relative humidity reaches a critical value, i.e., dewpoint, and small dewdrops start to aggregate, enabling the shaping of dewdrops through designing the hydrophilic pattern. It is clearly seen from Figure 2c that the transverse profiles of the dewdrops agree well with the hydrophilic pattern. Specifically, rectangular, cross, and H‐shaped dewdrops are condensed. Since the transverse profile of the dewdrop can be tailored almost arbitrarily, the EM response of dewdrop‐based unit cells can then be easily manipulated. Furthermore, as the condensation process continues further, dewdrops will become even more plump, and their thickness will increase. Therefore, the EM response of the dewdrop meta‐atom can be tuned by manipulating the condensation and evaporation processes by controlling the relative humidity and temperatures of the humid air and condenser, as shown in Figure 2b. Such a tunability of the dewdrop metasurface is verified in the following two examples.

### 2.2 Tunable Ultrabroadband Absorption Enabled by TDMs

**Figure 3:**
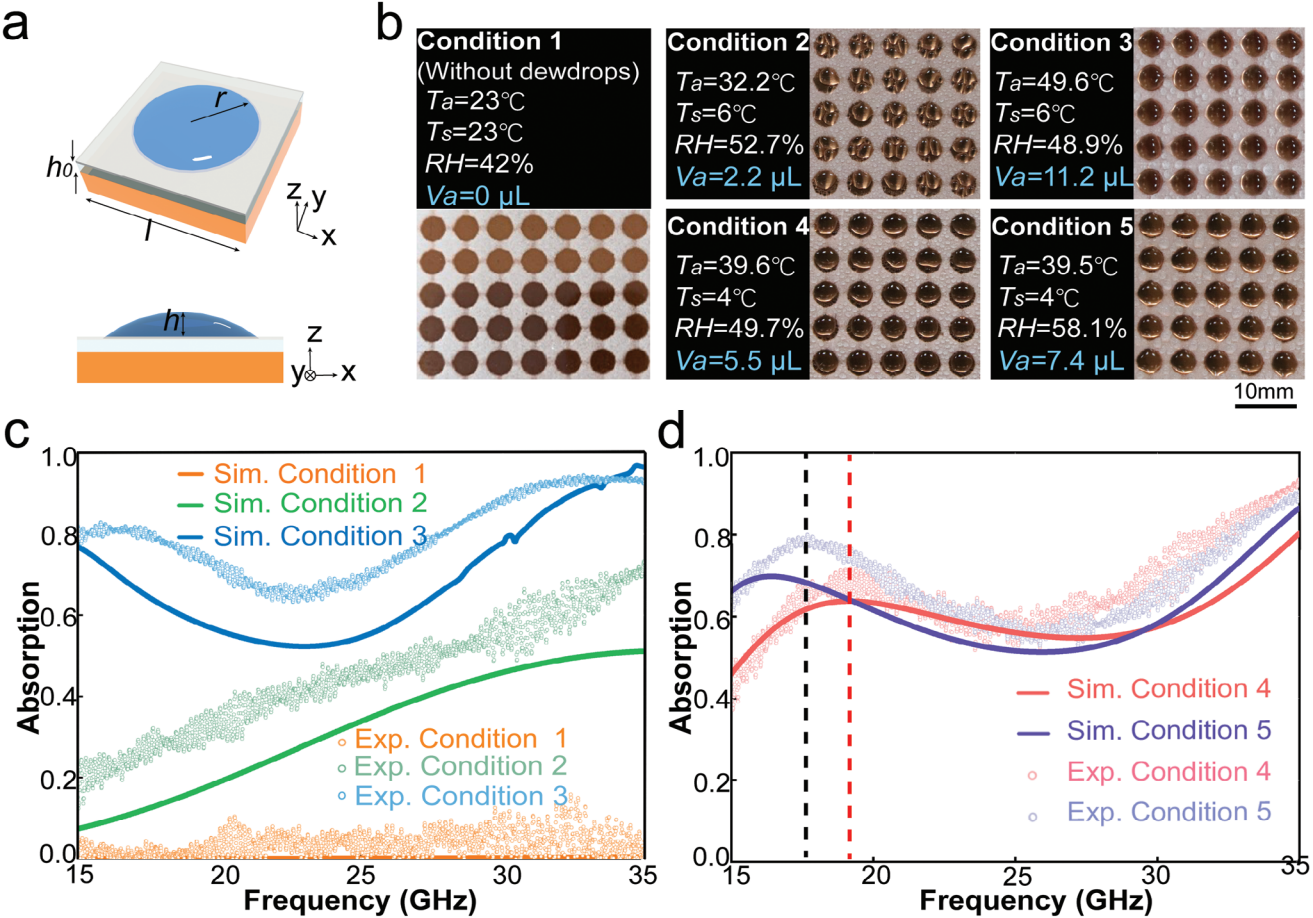
Tunable ultrabroadband absorption based on a TDM. a) Schematic of the meta‐atom of the TDMs. b) Photographs of ultrabroadband absorption TDMs under different ambient conditions, and the scale bar is 10 mm. c,d) Simulation and experimental results of the absorption spectrum c) under different temperature differences between *T*_a_ and *T*_s_ and d) different *RH*.

Water exhibits a large imaginary part of permittivity in microwave regimes and hence is a candidate material for conducting microwave absorbers. Here we design a real‐time TDM that can be tailored to exhibit different absorption characteristics by changing ambient conditions with the dewdrop preparation method mentioned above. **Figure** **3**a presents a meta‐atom of the TDM, which is composed of a layer of copper film (orange), a hydrophilic glass layer (gray) with hydrophobic patterns, and a spherical‐cap dewdrop (blue) on the hydrophilic region. The lattice constant of the meta‐atom is *l* = 5.5 mm. The thickness and the relative permittivity of the glass layer are *h*_0_ = 0.4 mm and *ε*_d_ = 6.5. The radius of the circular hydrophilic region is *r* = 2.2 mm and the dewdrop height *h* is determined by ambient vapor conditions. The calculated height *h* was based on the measured dewdrop volume on circular hydrophilic regions under different environmental conditions, and the simulation neglected tiny dewdrops condensed on hydrophobic regions. Here, dewdrops with circular profiles are chosen because spherical‐cap dewdrops can meet the impedance matching condition^[^ ^30^, ^31^ ^]^ by inducing magnetic resonance and suppressing the reflection for high absorption. The calculated H‐field distribution of the meta‐atom is shown in Figure S4 (Supporting Information) to explain the magnetic resonance of TDMs.

By adjusting the different ambient temperature (*T*_a_), substrate temperature (*T*_s_), and relative humidity (*RH*), we obtained a series of dewdrops with different morphologies, as shown in Figure 3b, under the same condensation duration of 1 hour. The small deviation between simulation and experiment results is attributed to tiny dewdrops condensed on hydrophobic regions and the gravity‐induced asymmetric shapes of the dewdrops. Under condition 1 (*T*_a_ = *T*_s_ = 23 °C, *RH* = 42%), temperature of water vapor does not reach the dewpoint, and hence there is no dewdrop on the glass. Compared to condition 1, conditions 2 and 3 possess an increasing temperature difference between *T*_a_ and *T*_s_ and the same *RH*. Dewdrops are formed under both conditions 2 and 3, and the average volume (*V*_a_) of each dewdrop is measured as 2.2 and 11.2 µL, respectively. For conditions 4 and 5, *T*_a_ and *T*_s_ are fixed at 40 and 4 °C, while *RH* increases from around 50% to around 60%. The *V*_a_ in conditions 4 and 5 are separately 5.5 and 7.4 µL. In order to observe the relationship between the environment and morphology of dewdrops, we record more dewdrop volume and condensing conditions in the Supporting Information. Evolutions of these dewdrops’ size over time (including conditions 2, 3, 4, and 5) are shown in Figure S5 and Table S2 (Supporting Information). From the experimental results shown in Figure 3b, we find that, through adjusting environmental conditions including *T*_a_, *T*_s_, and *RH*, we can govern the morphology of dewdrops on the substrate's surface, which is crucial in tuning the absorption efficiency of the TDMs.

The full structure of the TDMs supporting tunable ultrabroadband absorption consists of a 24 × 24 array of meta‐atoms, as shown in Figure 3a. We then examine how the absorption efficiency of the dewdrop metasurface is modulated under illumination of normal incidence and different ambient environmental conditions. The absorption results of the TDMs are calculated in the asymmetric background via the finite‐difference time‐domain (FDTD) method. In the simulation, unit cell boundary condition is adopted. Two Floquet ports are used to generate incident microwaves and measure the reflection and transmission coefficients, i.e., *r* and *t*. Then the absorption rate can be obtained as 1 − |*t*|^2^ − |*r*|^2^. The simulated and experimental absorption spectra of the TDMs are presented in Figure 3c under conditions 1, 2, and 3 in Figure 3b, where temperature differentials between *T*_a_ and *T*_s_ are 0, 26.2, and 43.6 °C, respectively. For case 1, where there is no dewdrop, the metasurface exhibits negligible absorption for EM waves from 15 to 35 GHz. On the contrary, for case 3, where dewdrops occupying the whole hydrophilic area appear, an average absorption of 0.79 (0.68) is achieved in the experiments (simulations) in the frequency band ranging from 15 to 35 GHz, and the measured (simulated) absorption efficiency is as high as 0.93 (0.96) at 35 GHz. The simulated and experimental absorption spectra of the TDMs under conditions 4 and 5 are illustrated in Figure 3d. The average absorptions under condition 4 (*RH* = 49.7%) and condition 5 (*RH* = 58.1%) are very close to each other due to the small change of the *V*_a_. Interestingly, a clear redshift of the low‐frequency absorption peak, i.e., from 19.1 GHz (red dashed line) to 17.7 GHz (black dashed line), is observed.

In this example, it is verified that by manipulating environmental conditions, ultrabroadband absorption with tunable absorbance can be attained through the TDMs. Additionally, TDM facilitates real‐time regulation of EM wave absorption rates. In high environmental humidity conditions, TDM exhibits enhanced spectral tuning responses. Moreover, within higher frequency bands, the absorption rate of this dewdrop metasurface increases more rapidly compared to lower frequency bands (see the Supporting Information, Figure S6). It is worth noting that the key factor directly affecting the tunable absorption is the volume of the dewdrop in the meta‐atom, which does not correspond to a certain environmental condition due to the combined influence of temperature and *RH* on the condensation and evaporation processes.

### 2.3 Tunable Binary‐Phase Gratings Enabled by TDMs

Binary‐phase grating^[^ ^59^ ^]^ is a special type of optical component that is formed with periodically arranged phase shifters with two different phases. The intensities of diffraction orders can be manipulated by changing the phase difference between the two phase shifters. Specifically, when such a phase difference equals π, only odd‐order diffractions appear.^[^ ^60^, ^61^ ^]^ Here, a tunable binary‐phase grating is constructed by utilizing TDMs, where the reflection phase difference can be tuned by changing the ambient conditions.

**Figure 4:**
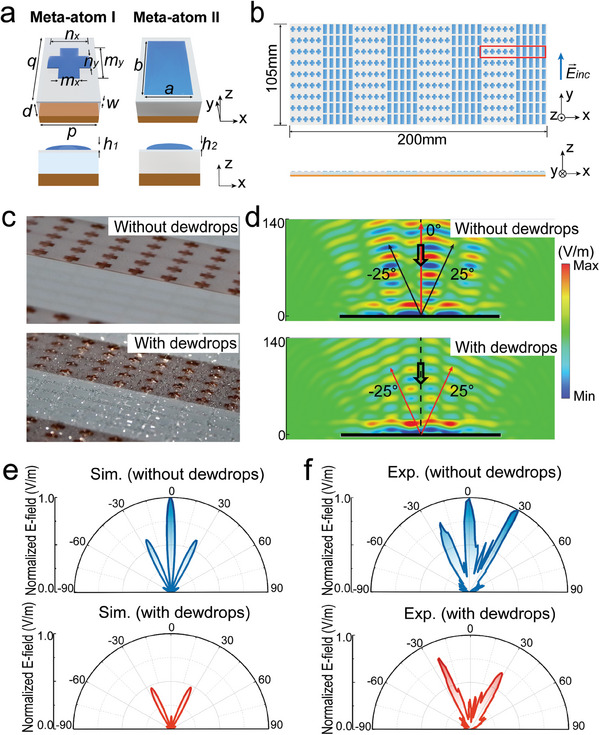
A tunable binary‐phase grating enabled by the TDM. a) Schematic of the meta‐atoms of the TDM. b) A tunable binary‐phase grating composed of meta‐atom I and II. c) Zoomed‐in view of fabricated TDM in two different states with and without dewdrops. d) Simulated electric‐field distribution of the TDM under normal incidences with *y*‐polarization at 14 GHz. Hollow arrows represent the incident beams. e) Simulated and f) measured far‐field scattering patterns of the TDM without and with dewdrops at 14 GHz.

Two types of dewdrop meta‐atoms are shown in **Figure** **4**a, which correspond to the phase shifter in traditional phase‐type gratings. Meta‐atom I, shown in the left panel, is composed of a layer of copper film (orange), a hydrophilic SiO_2_ layer (pale blue) with relative permittivity *ε*_I_ = 4.3, and a dewdrop in a cross hydrophilic region. Meta‐atom II, shown in the right panel, is composed of a layer of copper film (orange), a hydrophilic ceramic layer (gray) with relative permittivity *ε*_II_ = 8.9, and a dewdrop in a rectangular hydrophilic region. The lattice constants of Meta‐atom I and II in the *x* and *y* directions are *p* = 5.0 mm and *q* = 8.75 mm. The thickness of SiO_2_ and ceramic layer is *d* = 1.77 mm. The parameters of the cross hydrophilic region are *n_x_* = 3.8 mm, *n_y_* = 1.6 mm, *m_x_* = 1.8 mm, and *m_y_* = 4.2 mm. The parameters of the rectangular hydrophilic region are *a* = 2.9 mm, *b* = 7.4 mm. The height of Meta‐dewdrops I and II are separately *h*_1_ = 0.42 mm and *h*_2_ = 0.43 mm under the ambient conditions of *T*_a_ = 22.9 °C, *T*_s_ = 8.5 °C, and *RH* = 43.3%, which is referred to as condition 6. The real‐time ambient condition records are presented in the Supporting Information, Table S3. It is found that when there were no dewdrops on the metasurface, the reflectance phase difference, Δ*φ*, between the two meta‐atoms is $\frac{\pi }{2}$, and for both meta‐atoms, near‐unity reflectivity is achieved. However, when dewdrops condense on the metasurface under condition 6, the phase difference Δ*φ* equals π, and the reflectivity of two meta‐atoms decreases slightly. The detailed simulation results of reflectance coefficients for meta‐atoms I and II under TE‐polarized normal incidence at 14 GHz are shown in the Supporting Information, Table S4.

Tunable binary‐phase grating based on these two dewdrop meta‐atoms is illustrated in Figure 4b. The grating constant in the *x* direction is *L_x_* = 10*p* = 50 mm. Part views of the fabricated TDM are illustrated in Figure 4c. Due to the periodic phase distribution along *x*‐direction, the diffraction order obeys the diffraction equation

(1) $$L_{x}\sin\theta_{m}=m\lambda$$

where *θ*_m_ is the reflection angle of the *m*th reflection order and *m* can be integers. *λ* is the wavelength of the incident wave. We then verified the wavefront‐controlling functionalities of the TDM by numerical calculations and far‐field experiments. The simulated near‐field and far‐field electric‐field distributions of the TDM are shown in Figure 4d,e. The TDM is excited by a Gaussian beam at 14 GHz. The upper and lower panels show the electric‐field distributions of the meta grating without and with dewdrops, respectively. It is found from both Figure 4d,e. that without dewdrops on the metasurface, the 0th‐order and ±1st‐order reflection waves separately propagate toward directions of *θ*_0_ = 0° and *θ*_±1_ = ±25°, which are coincident with the theoretical prediction from Equation (1). However, when condition 6 is applied, dewdrops condense on the metasurface, and 0th‐order diffraction disappears while the ±1st‐order reflection is maintained. We also perform far‐field experiments to study the wavefront control of TDM under in environments. The setup of the far‐field measurements is shown in the supplement in the Supporting Information, Figure S7. The measured far‐field results at 14 GHz are shown in Figure 4f, which agrees well with the simulation results in Figure 4e, demonstrating that when there are dewdrops on the grating under condition 6, the 0th‐order diffraction is eliminated, and all the reflected energy is split to directions of *θ*_±1_.

In addition to these two particular states of the TDM grating, i.e., one without dewdrop and the other suppressing the 0th‐order diffraction, more states revealing the evolution of the 0th‐order diffraction from dominating to vanish can be realized by manipulating the morphology of dewdrops through designing the ambient condition. It can be inferred that the dewdrop height could decrease in less humid conditions, but the scattering pattern still keeps a portion of energy toward 0th‐order diffraction. The detailed far‐field simulation results are explained in the Supporting Information (see Figure S8). In this example, it is verified that by manipulating the ambient condition, TDMs, functioning as binary‐phase grating, can be tuned to generate diffraction beams with distinct intensity, demonstrating the potential of TDMs in wavefront control.

## 3 Conclusion

The formation and disappearance of dewdrops are common natural phenomena in life, yet our work here converts them into an artificial EM material with excellent switching functionality. To achieve this goal, not only the size but also the shape and lattice of the dewdrops should be tailored. Through the design of a pattern of hydrophilic and hydrophobic regions, we have successfully achieved this goal. The unprecedented switching mechanism based on condensation and evaporation can lead to distinct changes in functionalities, such as between zero absorption and near total absorption of microwaves.

Compared to previous water‐droplet metasurfaces,^[^ ^37^, ^38^ ^]^ in this work, we explored not only the microwave absorption properties but also the phase‐manipulation function via dewdrops of specific shapes, enabling the tailoring of the microwave wavefront by a TDM. Unlike previous active metasurface manipulation methods, the approach based on the condensation and evaporation processes to control the existence, disappearance, and morphological transformation of meta‐atoms and hence manipulate their EM responses is proposed for the first time. Different from active water‐based metasurfaces utilizing microfluidics^[^ ^40^ ^]^ and mechanical controlling technologies^[^ ^35^, ^36^, ^37^ ^]^ that rely on closed containers for water, this approach can be applied in environments without electrical, optical/thermal, or mechanical stimuli. For instance, it can be applied on walls, rocks, and even leaves to achieve amazing control and switching of microwaves through the natural condensation and evaporation of water that happen every day. This method is clean, environmentally friendly, and has a very low cost of fabrication. This concept can also be further extended to other frequencies and waves, such as optics and acoustics, impacting various disciplines. In addition, we note that although only a homogeneous dewdrop metasurface and a binary‐element dewdrop metasurface are demonstrated here, the concept of TDMs can be easily extended to phase gradient metasurface capable of more flexible manipulation of the wavefront by introducing more degrees of freedom. For example, dewdrops of meta‐atoms can be independently controlled to exhibit various phase shifts by assigning each meta‐atom a discrete condenser with tunable temperature. The TDMs also have some limitations, such as the absorption at microwave frequencies and the deformation of dewdrop shapes due to gravity.

In conclusion, we have proposed an intriguing dewdrop metasurface and introduced a novel regulating method of adjusting dewdrops through the condensation and evaporation processes. The transverse profiles of the dewdrops are realized by modifying the wettability distributions on the metasurface substrates. The condensation and evaporation of the dewdrops are controlled by changing the ambient temperature, the temperature of the substrate of the metasurface, and the relative humidity. It is worth noting that the dewdrop metasurface exhibits a high sensitivity to environmental conditions. Ambient temperature directly affects the surface nucleation rate,^[^ ^62^ ^]^ while relative humidity directly influences the growth and coalescence rates of the small droplets.^[^ ^63^ ^]^ Higher relative humidity and a larger temperature difference between the environment and the cold surface substrate enable the dewdrop metasurface to achieve faster EM responses. By using the TDMs, tunable ultrabroadband absorption and a tunable binary‐phase grating in the microwave frequency regime are demonstrated by both numerical simulations and microwave experiments. Such a method of controlling microwave metasurfaces based on the phase transformation process of water also applies to other liquids and might find potential applications in other frequency bands, such as optical frequencies, where water causes negligible propagation dissipation of light, leading to high‐efficiency devices. We also note that at higher frequencies, such as optical frequencies, the dewdrop metasurfaces require much smaller dewdrops of micrometer or nanometer scale, thus the dynamic controlling method based on the condensation and evaporation of dewdrops would be much more responsive and efficient. The proposed TDMs open a new route toward future economic, eco‐friendly smart tunable metamaterials.

## Conflict of Interest

The authors declare no conflict of interest.

## Supporting information

Supporting Information

## Data Availability

The data that support the findings of this study are available from the corresponding author upon reasonable request.
