# Supplementary material for: Dewdrop Metasurfaces and Dynamic Control Based on Condensation and Evaporation
Source: Adv Sci (Weinh). 2024 Aug 21;11(39):2404010. doi: 10.1002/advs.202404010 (PMC11497049; doi:10.1002/advs.202404010)
Supplement: Supplementary file 1 — Supporting Information [file ADVS-11-2404010-s001.docx]

**Supporting Information**

Title ：Dewdrop Metasurfaces and Dynamic Control Based on Condensation and Evaporation

Runqi Jia†, Yongxin Jing†, Hongchen Chu*, Ruwen Peng*, Mu Wang*, and Yun Lai*

1. The complex permittivity of water


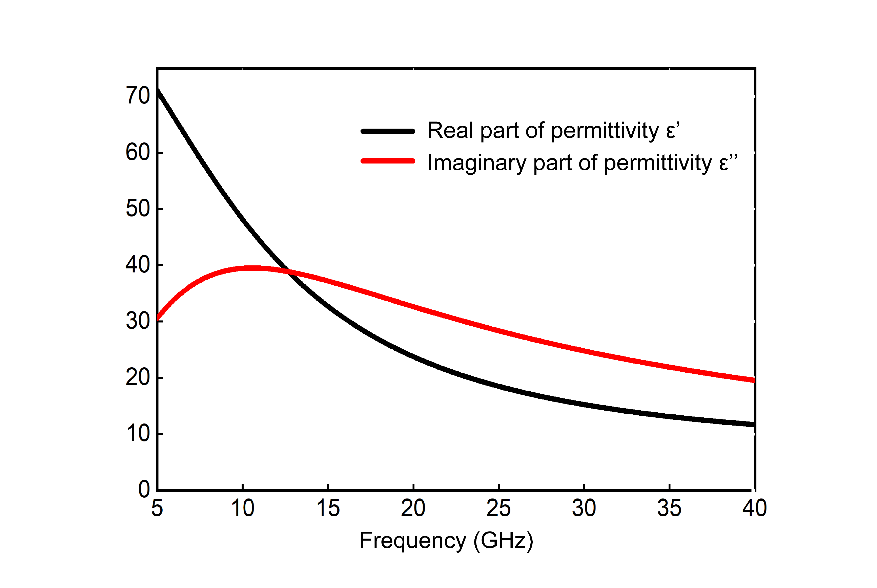


Figure S1. The permittivity of water over the frequency range 5-40 GHz at the temperature of 6℃. The result can be explained by three microwave relaxation processes ^[1]^ and numerical simulation matches well with experimental measurements. We note that, in this paper, dewdrop temperature is considered to be consistent with substrate condenser temperature (*T*_s_), which maintains an average of 6℃ during experiments.

1. Measured total dewdrop volumes on hydrophilic and hydrophobic surface

In order to verify the condensing ability of different surfaces (hydrophobic and hydrophilic) under the same condensing time duration as well as the same ambient environment (including ambient temperature, substrate cooling temperature, and ambient humidity), we measured the total dewdrops condensed on hydrophobic and hydrophilic surface, separately. Here, we note that the substrate temperature (*T*_s_) is 4℃, the ambient temperature (*T*_a_) is 18℃ and the ambient relative humidity (*RH*) equals 86%. Because of the wettability nature of the two types of surfaces, the initial nucleation density is higher than that on the hydrophobic surface ^[2]^. Thus, in the following experiments of TDMs, the hydrophilic region (SiO_2_ and ceramic) also exhibits a higher dewdrop condensation rate than the hydrophobic region (fluorosilane polymer) ^[3]^. We conducted three separate sampling measurements for each region, and Table S1 shows the detailed total dewdrop volumes on hydrophilic and hydrophobic surfaces. The volume results are measured by microliter syringes produced by GAOGE (item specification: 5μL). By withdrawing the total amount of dewdrops on hydrophilic or hydrophobic surfaces, the volume can be read according to values on syringes.


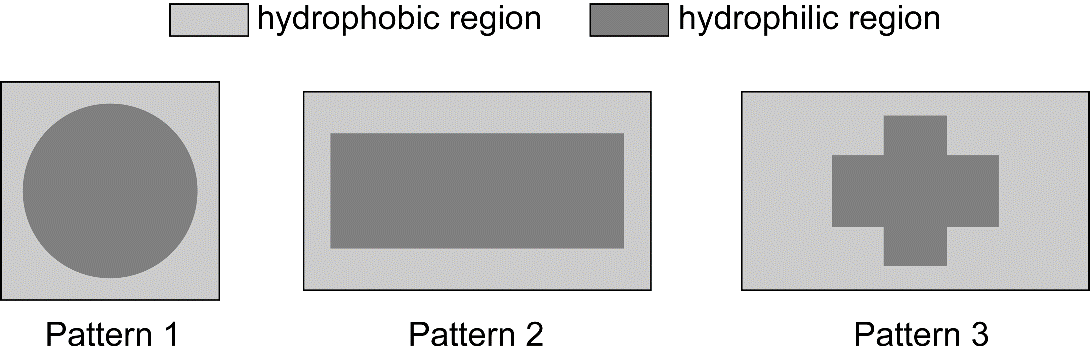


Table S1. Total volume of dewdrops on hydrophilic/hydrophobic regions of different patterns

| Condensing time (mins) | Sampling region | Total volume (μL) | | |
| --- | --- | --- | --- | --- |
|  |  | Measurement **1** | Measurement **2** | Measurement **3** |
| 60 | Pattern1 (hydrophobic) | 0.70 | 0.40 | 0.60 |
|  | Pattern1 (hydrophilic) | 1.98 | 1.96 | 2.03 |
| 40 | Pattern2 (hydrophobic) | 1.67 | 1.06 | 1.37 |
|  | Pattern2 (hydrophilic) | 2.00 | 1.93 | 2.12 |
| 40 | Pattern3 (hydrophobic) | 0.97 | 1.17 | 1.00 |
|  | Pattern3 (hydrophilic) | 1.70 | 1.57 | 1.86 |

1. Fabrication process of hybrid wettability metasurface substrate

The materials and tools used to engineer the hybrid wettability metasurface substrate are listed below.

Materials: cooper layer, hydrophilic dielectric layer [e.g. SiO_2_ glass (SiO_2_ ≥ 99.9%); soda-lime glass^[4]^(SiO_2_≈60-75%, Na_2_O≈10-25%, Ca_2_O≈5-15%; 95ceramic (Al_2_O_3_ ≥ 95%)], 3D-printed resinous pattern mask, hydrophobic Fluorosilane polymer^[5, 6]^(solvent: butyl acetate, solid solution content: 12%, static water-droplet contact angle>160°).

Tools: classical spray gun (valve caliber≥1mm, spray width≈10-25cm).


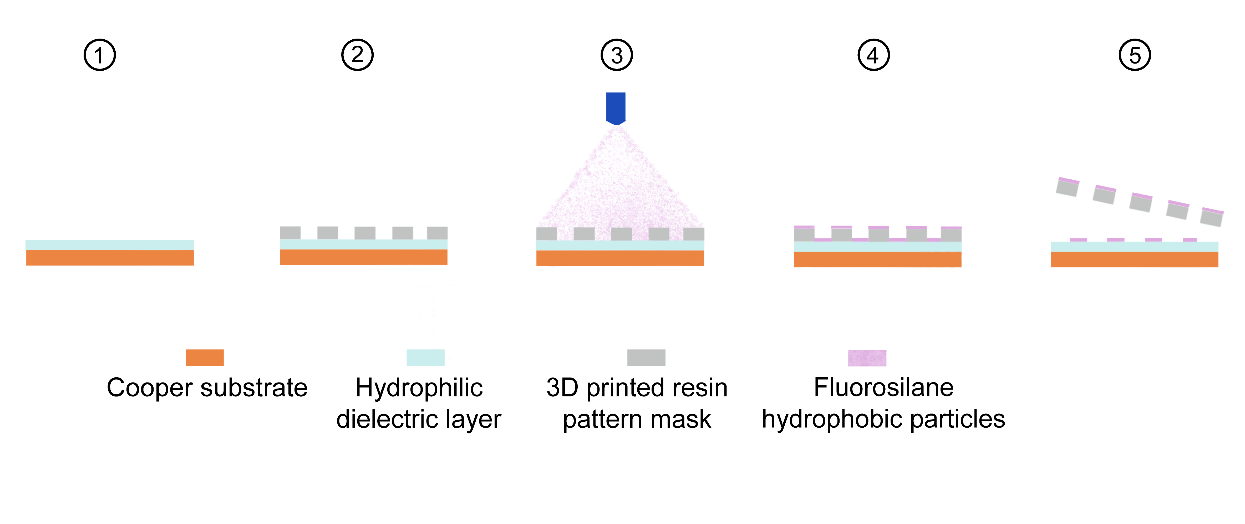


Figure S2. Fabrication procedure of engineering hybrid wettability on substrate surface.

Fabrication procedure:

1. Cover the copper plate with a flat hydrophilic dielectric layer and clean the front surface of the dielectric layer with 1% potassium hydroxide solution to remove oil contamination. Wipe dry with a lint-free cloth to ensure the surface remains clean and dry.

2. Place the 3D-printed resinous pattern mask onto the hydrophilic dielectric layer, exposing the regions that need to be processed into hydrophobic surfaces.

3. Use the spray gun to apply fluorosilane polymer nano-particles to the surface of the sample (spraying density≈120G⋅m^-2^, thickness≈15μm), ensure that the spray nozzle is perpendicular to the sample surface.

4. Place the sample in a drying oven at 120°C and dry for 30 minutes to allow the fluorosilane polymer coating to air dry and cure.

5. Remove the resinous pattern mask.

4. Real-time controlled dewdrop condensation and evaporation devices.

The temperature and humidity sensor/controller are all commercial components to realize the manipulation of humid air temperature. We reassembled these components to enable real-time external control, and information of device components as well as technical details are as follow:

*Components:* Intelligent humidity controller (model-Sieval HC-05B, measurement controlling range: 0-99% RH with an accuracy of ±2% RH); Capacitive humidity sensor probe (measurement range:0-99%RH); Intelligent temperature controller (model-WS-SM3A, measurement controlling range: -10-150℃ with an accuracy of ±2℃); Electrical heating wires (5 meters long with the power of 50W); Commercial temperature sensor (measurement range:0-100℃); Semiconductor condenser (model-double core /quadruple copper tubes, general power 100W).


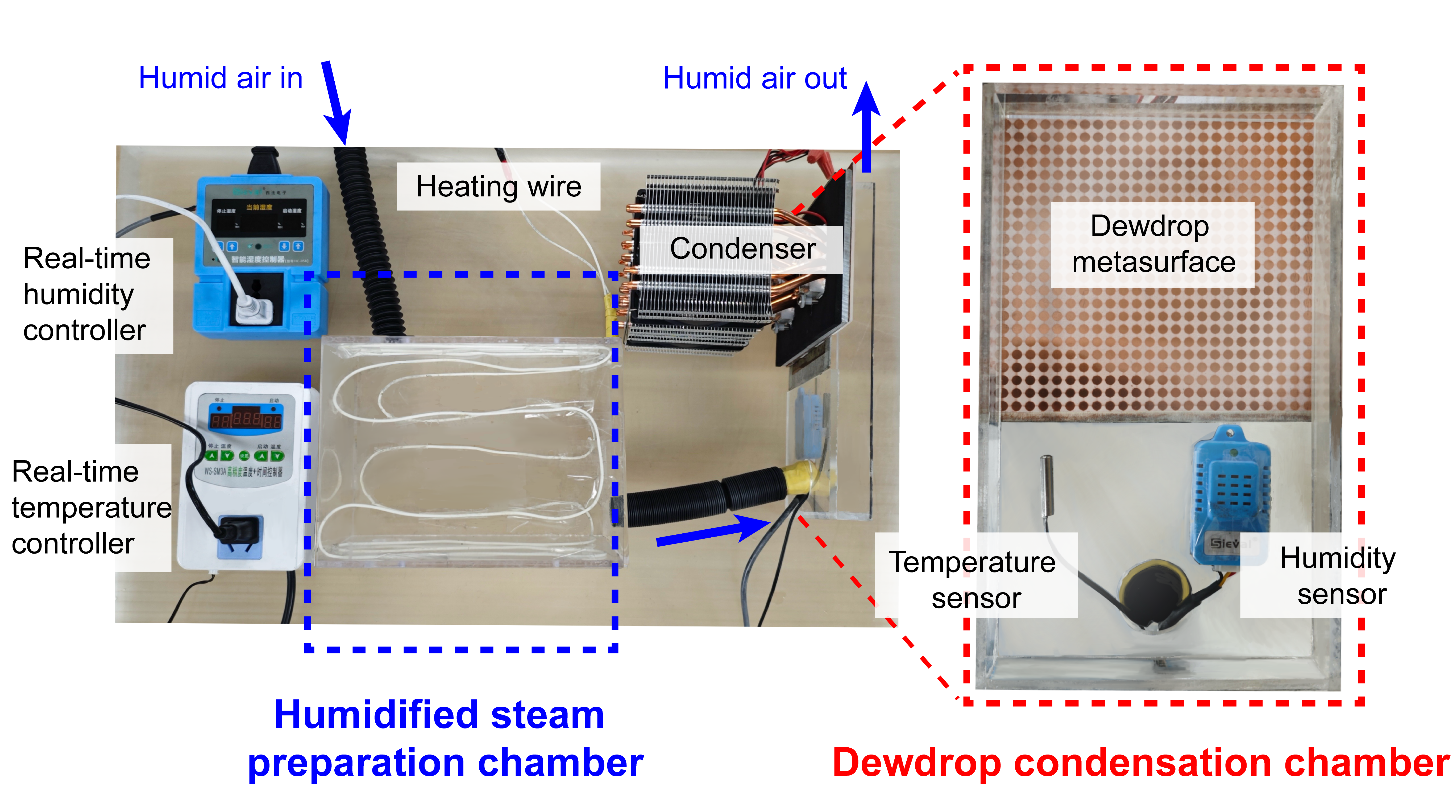


Figure S3. The side view of real-time controlled dewdrop condensation and evaporation devices. (left panel) Overall picture of controlling devices. (right panel) front view of dewdrop condensation controlling chamber.

*Chamber design:* The walls of humidified steam preparation chamber and the dewdrop condensation chamber are made of acrylic sheets (PMMA). In humidified steam preparation chamber, there retained two holes for humid air in and transport. For the dewdrop condensation chamber, a square area was reserved on the rear wall of the chamber to accommodate the metasurface substrate in direct contact with the condenser. The front wall of the condensation chamber was replaced with a thin plastic film instead of PMMA, serving as a window to observe the condensation of dewdrops on the metasurface substrate.

*Controlling process:* Electrical heating wires are wound parallelly inside the humid steam preparation chamber, with their control switch connected to the external temperature controller. The humid steam control switch is linked to the external humidity controller, allowing regulation of the water vapor inflow by toggling the switch. Temperature and humidity sensor probes are placed inside the dewdrop condensation chamber to monitor real-time temperature and humidity data. These data are transmitted to external temperature and humidity controllers, enabling feedback and adjustment of the environmental conditions within the condensation chamber.

1. Numerical analysis of dewdrop metasurface absorption

To achieve broadband tunable absorption, spherical dewdrop unit structures induce magnetic resonance ^[7]^ in the lower frequency range. Condensing periodic dewdrops on a metasurface substrate introduces a two-dimensional diffraction grating effect ^[8, 9]^, resulting in another absorption peak in the higher frequency range. The absorption results of the unit cell are calculated in the asymmetric background via FDTD methods


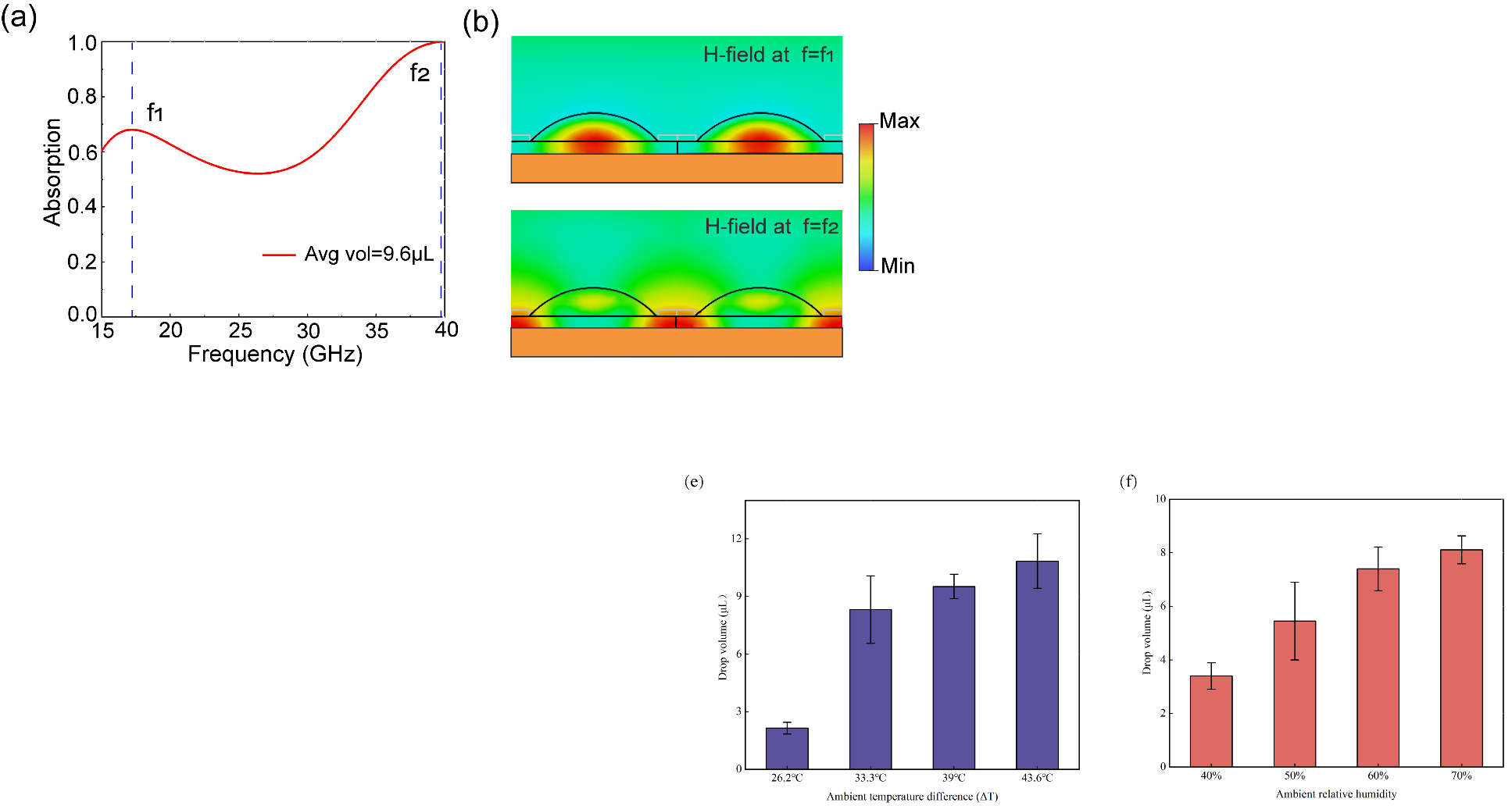


**Figure S4.** a) Simulation results of the absorption spectrum of dewdrop metasurface when the average volume (*Va*) of dewdrops equals 9.6μL. The blue dashed lines mark the absorption peak *f*_1_=17.2 GHz and *f*_2_=39.6 GHz, respectively. b) Side view of magnetic distribution H_x_ at the absorption peaks *f*_1_ and *f*_2_.

1. Experimental data of condensed dewdrop volume

In the experiment of dewdrop metasurface absorption, we set and compared a series of environmental conditions to explore the condensing process. We arrange two experimental groups, one of which maintains the same relative humidity (*RH*) but has different temperature *ΔT*. (In this experiment, the temperature of the condenser and substrate (*T*_s_) remains constant, we varied the temperature of the ambient vapor (*T*_a_), thereby changing the temperature difference *ΔT* between the substrate and environment). The other one keeps the same ambient vapor temperature but varies in relative humidity (*RH*). It is observed that the dewdrop volume increases when the temperature differences enlarge or under a more humid environment at the same condensing time. The volumes are measured by microliter syringes produced by GAOGE (item specification: 25μL).


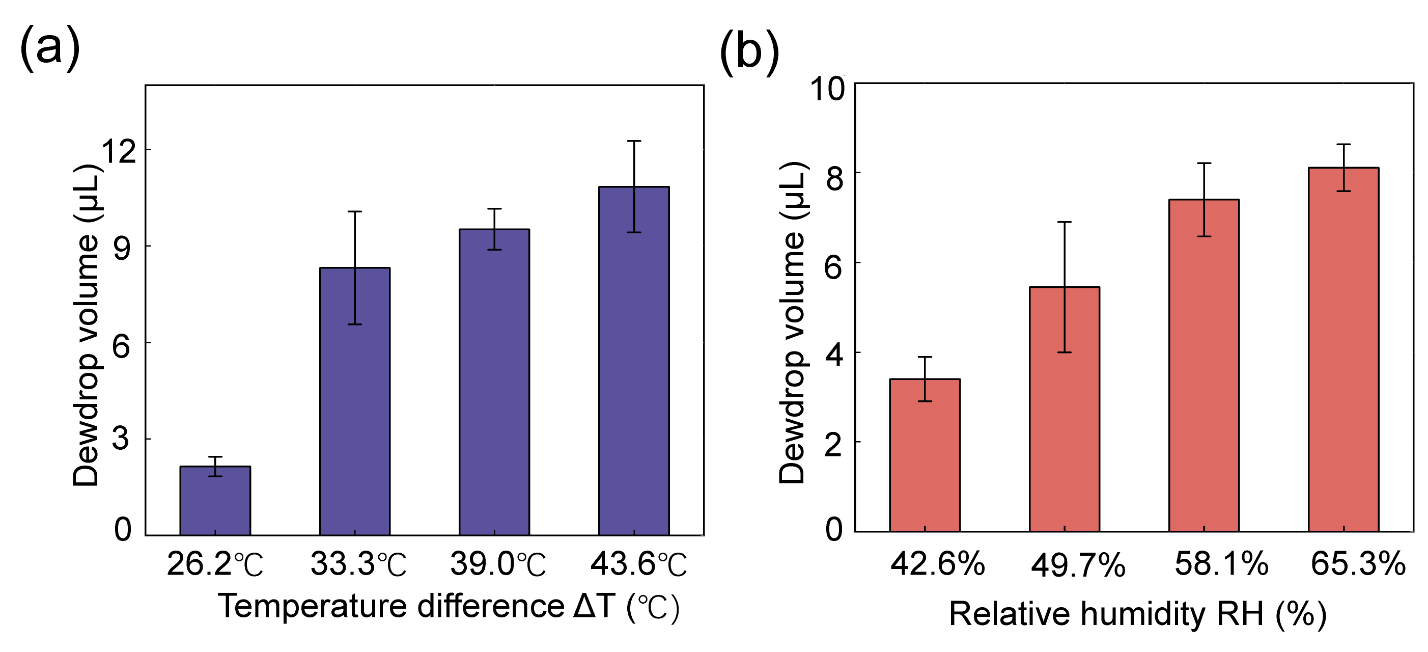


**Figure S5.** a) The average dewdrop volume under different ambient temperature conditions. The average *RH* of this group is 50±3%. b) The average dewdrop volume under different relative humidity conditions. The average ambient temperature *T*_a_ maintains (40±0.5) ℃

1. Experimental data of ambient temperature and relative humidity

We record the real-time temperature and relative humidity during the condensing process to observe the relationship between environmental conditions and dewdrop volume. The experimental group labeled here corresponds to the two groups mentioned in Figure S5, respectively. The first four groups are designed to maintain the average volume around 50% and record the influence induced by the average ambient temperature change. The last four groups keep the same average ambient temperature around 40℃ but vary the relative humidity during condensation.

Table S2. Real-time temperature ($T_{a}$) and relative humidity (*RH*) of two experimental groups

| Experiment groups | | Condensing time (minute) | | | | | | | | | | | | | |
| --- | --- | --- | --- | --- | --- | --- | --- | --- | --- | --- | --- | --- | --- | --- | --- |
|  |  | 10 mins | | 20 mins | | 30 mins | | 40 mins | | 50 mins | | | 60 mins | | |
| Avg  *T_a_* (℃) | Avg *RH*(%) | *T_a_* (℃) | *RH* (%) | *T_a_* (℃) | *RH* (%) | *T_a_* (℃) | *RH* (%) | *T_a_* (℃) | *RH* (%) | *T_a_* (℃) | *RH* (%) | *T_a_* (℃) | | *RH* (%) |  |
| 32.2℃ 52.7% | | 32.8 | 45.3 | 34.0 | 49.1 | 34.1 | 54.4 | 30.4 | 46.3 | 30.5 | 61.4 | 31.2 | | 59.7 |  |
| 39.3℃ 48.5% | | 38.8 | 32.8 | 41.6 | 30.3 | 39.1 | 55.0 | 37.8 | 60.3 | 38.0 | 55.9 | 40.5 | | 56.5 |  |
| 45.0℃ 49.1% | | 43.5 | 43.8 | 47.5 | 41.4 | 46.4 | 48.1 | 46.0 | 56.2 | 46.7 | 56.7 | 40.7 | | 48.5 |  |
| 49.6℃ 48.9% | | 45.9 | 50.0 | 44.5 | 49.1 | 46.5 | 43.7 | 54.7 | 49.3 | 54.0 | 50.1 | 52.0 | | 51.2 |  |
| 39.9℃ 42.6% | | 39.1 | 37.9 | 38.9 | 39.5 | 42.0 | 47.5 | 40.7 | 45.1 | 38.6 | 39.1 | 40.3 | | 46.6 |  |
| 39.6℃ 49.7% | | 41.1 | 45.0 | 39.5 | 48.5 | 38.1 | 46.0 | 38.9 | 54.0 | 40.8 | 47.0 | 39.0 | | 57.7 |  |
| 39.5℃ 58.1% | | 38.4 | 54.9 | 41.8 | 59.5 | 38.6 | 68.3 | 40.2 | 52.8 | 38.5 | 56.0 | 39.7 | | 57.2 |  |
| 39.9℃ 65.3% | | 41.0 | 67.8 | 38.3 | 60.6 | 38.3 | 62.8 | 40.5 | 63.3 | 41.4 | 66.8 | 39.6 | | 70.5 |  |

8. The absorption performance of dewdrop metasurface during the growth and coalescence of incipient droplets

Here, we arranged two different groups, which has separate environmental relative humidity (*RH*), to demonstrate the effect of humidity on absorption rate. The relative humidity of group A and B are (60±3)% and (80±3)%, respectively. The temperature of the metasurface substrate was (8±2)℃, and the ambient temperature was (25±2)℃ in both group A and B. We captured the incipient droplets appearance in real-time with camera in left panel of figure S6a and S6b. The real time absorptivity under different frequencies and separate humidity conditions are shown in the right panel of figure S6a and S6b.


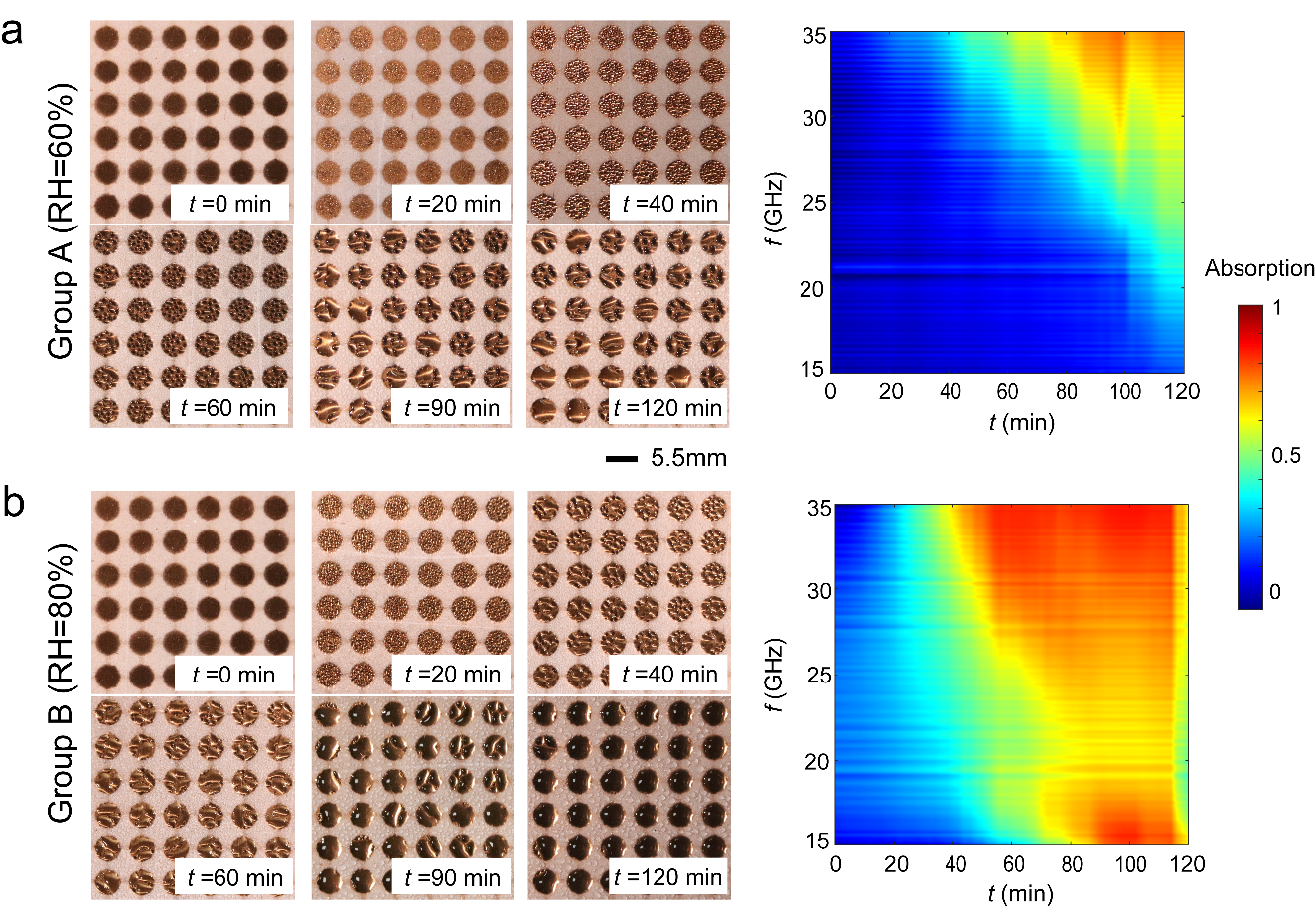


Figure S6. The zoom in views of metasurface in real time and heat map of absorption under 15-35GHz and different relative humidity around a). 60% and b). 80%. All of the scale bars are 5.5mm.

By comparing the absorption in figure S6a) and S6b), the dewdrop metasurface took 40 minutes to achieve an absorption rate of 40% at 35 GHz in group A, whereas group B required only about 20 minutes. At the 20 GHz frequency band, group B achieved a 50% absorption rate in just 40 minutes, while group A required a relatively longer time. Therefore, under high environmental humidity, TDMs can achieve faster spectral tuning responses. It is also found from both figure S6a and S6b that in higher frequency band, the increase in absorption rate of this dewdrop metasurface is faster than that in lower frequency band. Compared with left pictures, the key point for observing low-frequency absorption peak during condensation process is the formation of spherical-cap dewdrop atoms on hydrophilic regions. It should be noted that the sudden decline of absorption at 120 minutes in right panel of figure S6b was because the falling of plump dewdrops induced by gravity. (Defects on the dewdrop metasurface are not displayed in Figure S6b))

Here we emphasize that the time of formation of the dewdrop metasurfaces is dependent on the scale of the artificial dewdrops. For microwaves, the functioning dewdrops are at the millimeter scale, so the time is relatively long. But at optical frequencies, the functioning dewdrops are at micrometer or nanometer scale, thus the time could be significantly shortened.

1. The ambient condition of binary-phase TDMs during condensation

Here, we present the condensing time as well as the ambient environment condition (temperature and relative humidity) during the condensation procedure of binary-phase TDMs

Table S3. The condensing environment of binary-phase TDMs

| Time | *T_s_* (℃) | *T_a_* (℃) | *RH* (%) |
| --- | --- | --- | --- |
| 0min | 15 | 22.3 | 46.3 |
| 35mins | 12 | 23.1 | 41.9 |
| 56mins | 6.3 | 22.5 | 44.0 |
| 74mins | 6.9 | 23.0 | 43.2 |
| 91mins | 8 | 23.1 | 42.9 |
| 111mins | 6.6 | 23.1 | 42.9 |
| 150mins | 4.6 | 23.1 | 41.8 |

1. Reflectance coefficient for the meta-atoms I and II of binary-phase gratings enabled by TDMs

Here, we show the calculated reflectance coefficient of meta-atom I and II by FDTD methods under normal incidence at 14GHz. When dewdrops condensed on TDMs substrate, the incident wave energy was partly absorbed by dewdrops, which resulted in reduced reflectivity. Meanwhile, the magnetic resonance induced by dewdrops altered the reflectance phase difference from π/2 to π.

Table S4. Reflectance coefficient for the meta-atom I and II

|  | Without dewdrops | | With dewdrops | |
| --- | --- | --- | --- | --- |
|  | $\left\vert r \right\vert$ | $\varphi$ | $\left\vert r \right\vert$ | $\varphi$ |
| Meta-dewdrop I | 1.00 | 84° | 0.59 | 21° |
| Meta-dewdrop II | 1.00 | -10° | 0.51 | -160° |

1. Setup of the far-field measurement


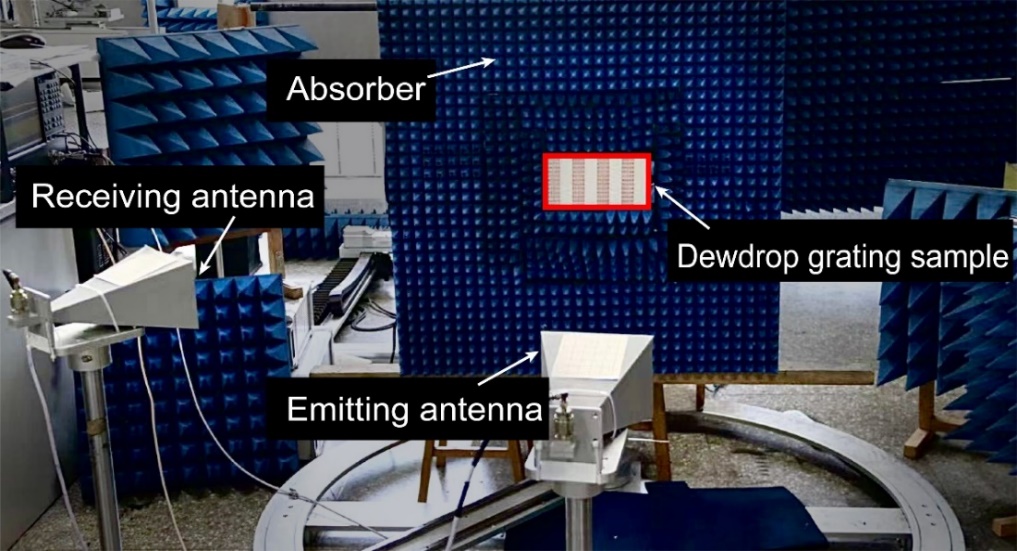


**Figure S7.** Setup of the far-field experiment. An emitting horn antenna is placed to generate the normal incident beam while the receiving antenna is arranged on a circular track, which can rotate around the dewdrop meta grating sample and measure the E-field at each polar angle in the *x-z* plane. The emitting and receiving antenna are connected to the network analyzer and the absorbers surrounding the sample are mounted to absorb scattering waves from other ambient objects.

1. The calculated far-field pattern of TDM grating under different dewdrop morphology

The far-field scattering patterns under different morphology of TDM grating are shown in Figure S8. We arrange different dewdrop heights to represent three dewdrop morphology states under different environmental conditions (0mm, 0.12mm, and 0.43mm, respectively).


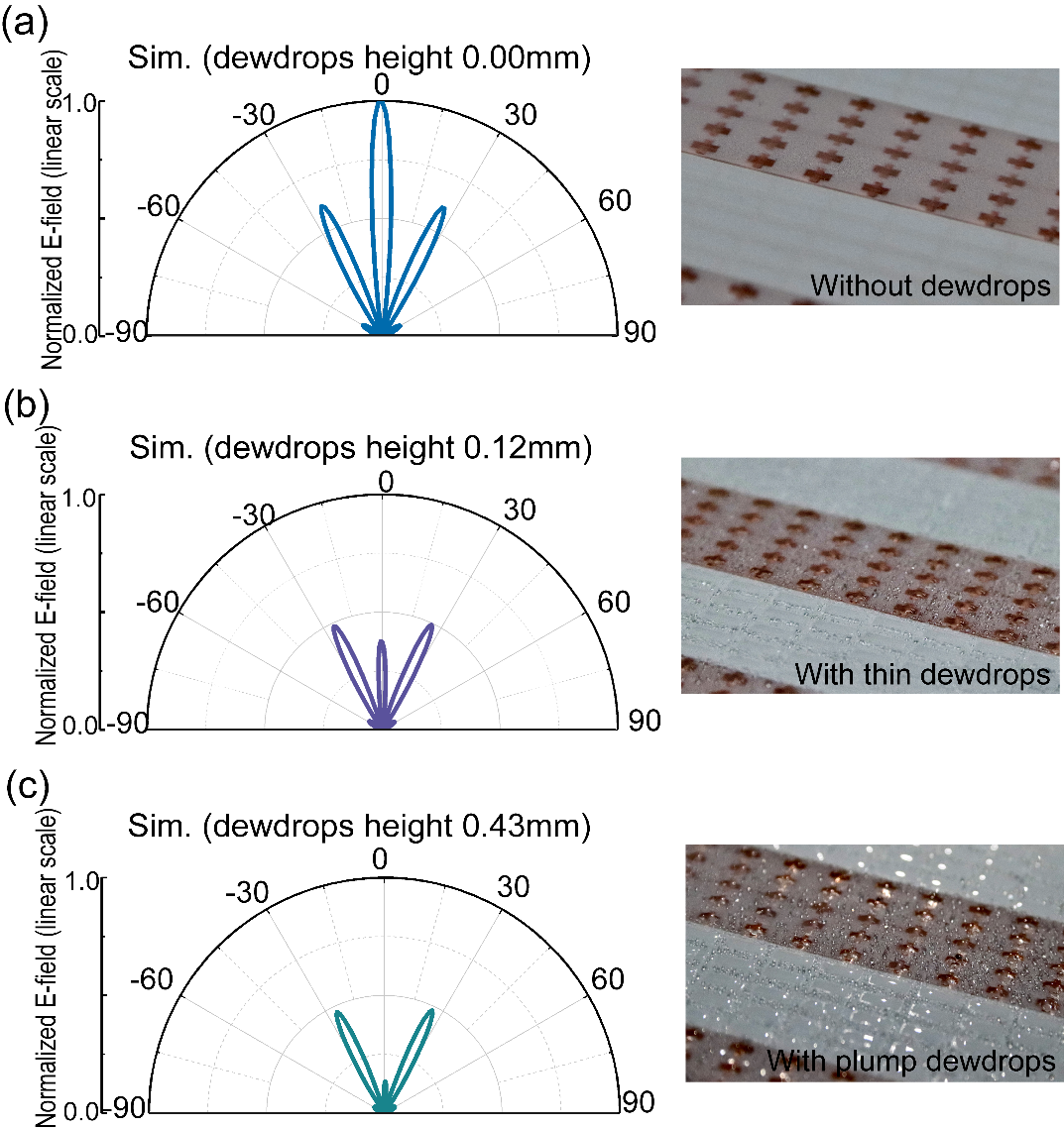


**Figure S8.** The simulation results of different TDM grating states. a) without dewdrops, b) with thin dewdrops, and c) with plump dewdrops. The left panel of (a-c) shows the normalized E-field scattering pattern of three states and the corresponding zoomed-in view on the right panel.

References

[1] W. J. Ellison, *J. Phys. Chem. Ref. Data* **2007**, 36.

[2] H. Cha, H. Vahabi, A. Wu, S. Chavan, M. -K. Kim, S. Sett, S. A. Bosch, W. Wang, A. K. Kota, N. Miljkovic, *Sci. Adv.* **2020**, 6, eaax0746

[3] B. Peng, X. Ma, Z. Lan, W. Xu, R. Wen, *Inter. J. Heat Mass Tran.* **2015**, 83, 27.

[4] F. Lisco, A. Shaw, A. Wright, J. M. Walls, F. Iza, *Sol. Energy* **2017**, 146, 287.

[5] J.-D. Brassard, D. K. Sarkar, J. Perron, *Appl. Sci.* **2012**, 2, 453.

[6] E.-C. Cho, C.-W. Chang-Jian, H.-C. Chen, K.-S. Chuang, J.-H. Zheng, Y.-S. Hsiao, K.-C. Lee, J.-H. Huang, *Chem. Eng. J.* **2017**, 314, 347.

[7] J. Zhao, S. Wei, C. Wang, K. Chen, B. Zhu, T. Jiang, Y. Feng, *Opt. Express* **2018**, 26, 8522.

[8] Y. Qu, Q. Li, H. Gong, K. Du, S. Bai, D. Zhao, H. Ye, M. Qiu, *Adv. Opt. Mater.* **2016**, 4, 480.

[9] Q. Song, W. Zhang, P. C. Wu, W. Zhu, Z. X. Shen, P. H. J. Chong, Q. X. Liang, Z. C. Yang, Y. L. Hao, H. Cai, H. F. Zhou, Y. Gu, G.-Q. Lo, D. P. Tsai, T. Bourouina, Y. Leprince-Wang, A.-Q. Liu, *Adv. Opt. Mater.* **2017**, 5, 1601103.
